# Rapamycin-mediated lifespan increase in mice is dose and sex dependent and metabolically distinct from dietary restriction

**DOI:** 10.1111/acel.12194

**Published:** 2014-02-09

**Authors:** Richard A Miller, David E Harrison, Clinton M Astle, Elizabeth Fernandez, Kevin Flurkey, Melissa Han, Martin A Javors, Xinna Li, Nancy L Nadon, James F Nelson, Scott Pletcher, Adam B Salmon, Zelton Dave Sharp, Sabrina Van Roekel, Lynn Winkleman, Randy Strong

**Affiliations:** 1 Department of Pathology and Geriatrics Center, University of MichiganAnn Arbor, MI, 48109, USA; 2 The Jackson LaboratoryBar Harbor, ME, 04609, USA; 3 Geriatric Research, Education and Clinical Center and Research Service, South Texas Veterans Health Care SystemSan Antonio, TX, 78229, USA; 4 Department of Psychiatry, The University of Texas Health Science Center at San AntonioSan Antonio, TX, 78229, USA; 5 Division of Aging Biology, National Institute on AgingBethesda, MD, 20892, USA; 6 Department of Physiology and Barshop Institute for Longevity and Aging Studies, The University of Texas Health Science Center at San AntonioSan Antonio, TX, 78229, USA; 7 Department of Molecular and Integrative Physiology and Geriatrics Center, University of MichiganAnn Arbor, MI, 48109, USA; 8 Barshop Institute for Longevity and Aging Studies, University of Texas Health Science Center San AntonioSan Antonio, TX, 78245, USA; 9 Department of Molecular Medicine, University of Texas Health Science Center San AntonioSan Antonio, TX, 78245, USA

**Keywords:** aging, caloric restriction, glucose, IGF-1, insulin, longevity, mouse, mTOR, rapamycin, xenobiotic metabolism

## Abstract

Rapamycin, an inhibitor of mTOR kinase, increased median lifespan of genetically heterogeneous mice by 23% (males) to 26% (females) when tested at a dose threefold higher than that used in our previous studies; maximal longevity was also increased in both sexes. Rapamycin increased lifespan more in females than in males at each dose evaluated, perhaps reflecting sexual dimorphism in blood levels of this drug. Some of the endocrine and metabolic changes seen in diet-restricted mice are not seen in mice exposed to rapamycin, and the pattern of expression of hepatic genes involved in xenobiotic metabolism is also quite distinct in rapamycin-treated and diet-restricted mice, suggesting that these two interventions for extending mouse lifespan differ in many respects.

## Introduction

Rapamycin, an inhibitor of mTOR kinase, increases lifespan of genetically heterogeneous UM-HET3 mice when given at a dose of 14.7 mg kg^−1^ in food from either 9 or 20 months of age (Harrison *et al*., 2009; Miller *et al*., 2011). Genetic or pharmacologic inhibition of mTOR has been shown to increase lifespan of yeast (Kaeberlein *et al*., 2005; Powers *et al*., 2006), worms (Vellai *et al*., 2003) and flies (Kapahi *et al*., 2004), suggesting that the link between TOR and aging may have deep evolutionary roots. Rapamycin also extends mouse lifespan (i) when given as a brief (6 week) treatment to aged C57BL/6 mice (Chen *et al*., 2009), (ii) when given by subcutaneous injection to female mice carrying the tumorigenic HER-2/neu transgenic mice (Anisimov *et al*., 2010), or (iii) when administered by injection to female inbred 129/Sv mice (Anisimov *et al*., 2011). Genetic knockout of S6 kinase 1, one of the mediators of mTORC1 action, has been shown to increase lifespan of female (but not of male) C57BL/6 mice (Selman *et al*., 2009), consistent with the rapamycin studies. Although rapamycin can block function of both mTOR complexes, differential inactivation of mTORC1 via hemizygous deletion of both mTOR and mLST8 extends lifespan of female (but not of male) mice on a background containing a mixture of C57BL/6 and 129S5 genes (Lamming *et al*., 2012), suggesting that effects on TORC1 may be particularly germane to control of aging and/or late-life lethal disease.

The mechanism or mechanisms through which rapamycin increases mouse lifespan are still uncertain. Inhibitors of mTOR function can block tumor growth directly (Hidalgo & Rowinsky, 2000), and it is plausible that direct effects on tumors may contribute to lifespan effects in mice, in which tumors are the most frequent class of lethal illness (Miller & Chrisp, 2002; Lipman *et al*., 2004). Enteric-released rapamycin-treated UM-HET3 mice, however, show decelerated rates of change in a wide range of age-related physiological and pathological endpoints (Wilkinson *et al*., 2012), suggesting that the drug may be inhibiting at least some aspects of the aging process in addition to any direct effects it may have on cancer cells. An anti-aging effect is also the most likely pathway for lifespan extension in worms and flies, in which neoplastic disease is uncommon as a cause of death.

Our reports on rapamycin emerged from a study conducted by the Interventions Testing Program (ITP; http://www.nia.nih.gov/research/dab/interventions-testing-program-itp), in which drugs are tested for mouse longevity effects at three institutions using a shared protocol (Miller *et al*., 2007; Nadon *et al*., 2008). A priori, results of drug efficacy emerging from testing single doses of each test agent are likely to underestimate the optimal effect of the drugs tested, because it is unlikely that the dose chosen for the initial test is at the peak of the dose/response curve. To test whether optimal effects may emerge from doses higher, or lower, than the one used in the initial study, we exposed cohorts of UM-HET3 mice to rapamycin at three doses, over a ninefold range, as a step toward determining the optimal dose for lifespan prolongation in mice. We also explored the idea (Kaeberlein & Kennedy, 2009) that the lifespan extension caused by lifelong dietary restriction (DR) might also be due largely to inhibition of mTOR function, by comparing features of DR mice to those of rapamycin-treated mice.

## Results

**Figure 1:**
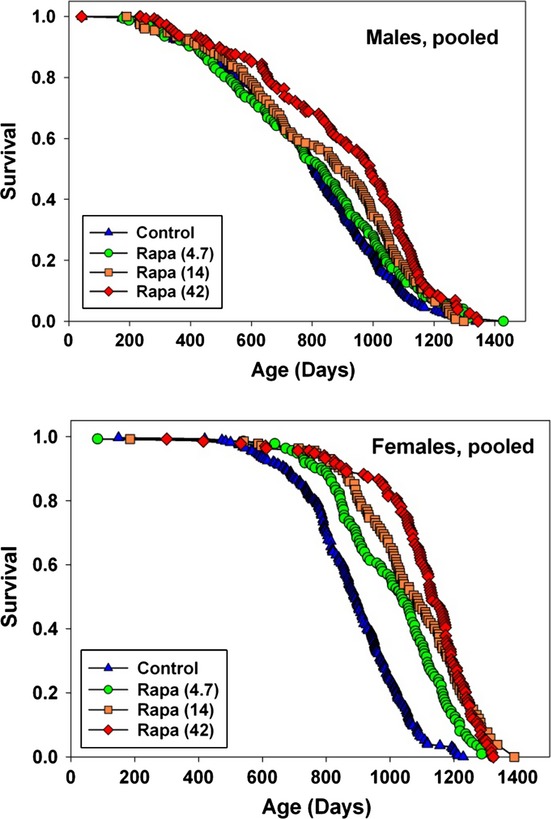
Survival curves at varying doses of rapamycin. Survival curves for male (top) and female (bottom) mice exposed to varying doses of rapamycin from 9 months of age. Data are pooled across the three test sites. At the time of analysis, fewer than 1% of the mice were still alive. Significance tests, median age, age of 90% mortality, and numbers of mice are shown in Table 1.

Mice were given food containing encapsulated rapamycin at one of three doses at age 9 months and followed until death to produce the survival data shown in Fig. 1, which pools results from all three sites. All three doses extended lifespan in females, with higher effects seen at the higher doses. The two highest doses also extended lifespan in male mice. Table 1 collects statistics from these pooled survival tables. The highest tested rapamycin dose increased median lifespan by 23% in males and 26% in females. Maximal lifespan, evaluated according to Wang *et al*., (2004) as the proportion of mice still alive at the age of 90% mortality in the joint lifespan distribution, was increased significantly (*P* < 0.001) by each of the three rapamycin doses in females and by the two higher doses in males (*P* = 0.006 and *P* = 0.001). Cox regression, stratified by site, showed a significant effect of dose (*P* < 0.001) for each sex and a significant difference between males and females, also at *P* < 0.001.

**Table 1:** Survival statistics pooled across sites

	Median	Median increase	Log-rank *P*-value	*N*	90th %ile age	90th %ile increase	Wang/Allison
Males							
Controls	807			300	1094		
Rapamycin (4.7)	834	3	0.19	156	1162	6	0.23
Rapamycin (14)	909	13	0.0015	156	1180	8	0.003
Rapamycin (42)	992	23	<0.0001	156	1185	8	0.004
Females							
Controls	896			280	1159		
Rapamycin (4.7)	1043	16	<0.0001	136	1218	5	<0.0001
Rapamycin (14)	1086	21	<0.0001	136	1285	11	<0.0001
Rapamycin (42)	1132	26	<0.0001	136	1282	11	<0.0001

Comment: median increase and 90th percentile increase are with respect to the corresponding values in the pooled controls of the same sex. Log-rank *P*-values were calculated with stratification by site. Wang/Allison test *P*-values (Wang *et al*., 2004) used the Fisher’s exact test to compare the counts of live and dead mice, added across the three test sites, at the site-specific age of 10% survival for the relevant joint distribution.

We also evaluated rapamycin effects at each of the three test sites independently, while recognizing that the lower number of mice at each site leads to a major loss of statistical power. Figure S1 (Supporting information) shows survival curves at each of the three test sites, and Table S1 (Supporting information) shows the corresponding statistics for each site. Rapamycin at 42 ppm led to a significant improvement in survival, in males, at each site, as assessed by the log-rank procedure, with median lifespan increasing between 7% (at TJL) and 31% (at UT). The effect of 14 ppm was more variable, with effects ranging from a 21% increase at UT to a 10% decline in median lifespan at TJL; as noted above, the primary endpoint, that is, a site-stratified log-rank test in the pooled data set, was significant at *P* = 0.002 in males, confirming our earlier report at this dose (Miller *et al*., 2011). Rapamycin at 4.7 ppm was not effective in increasing male lifespan in the pooled data set, although it did lead to significant lifespan extension (16%, *P* = 0.02) at UM. In females, even the lowest dose of rapamycin led to a significant survival benefit by the log-rank test and a significant result in the Wang/Allison test at 90th percentile mortality, at each site. The highest dose led to increases in median of 20%, 22%, and 31% at the three sites, representing good consistency for lifespan experiments of this kind.

Rapamycin concentrations were evaluated by HPLC–tandem mass spectroscopy (Livi *et al*., 2013) in whole blood taken from a separate set of UM-HET3 mice exposed to rapamycin, at UT, for 5 months starting at 3 or 4 months of age. Blood concentrations were 7, 16, and 80 ng mL^−1^ for females at rapamycin doses of 4.7, 14, and 42 ppm, respectively, and the corresponding values in males were 6, 9, and 23 ng mL^−1^, with *N* = 4–8 mice in each group (Fig. S2, left panel). A two-factor anova (sex and rapamycin dose) showed significant effects of sex (*P* = 0.007), rapamycin (*P* = 0.003), and interaction (*P* = 0.03), indicating that blood levels of rapamycin were higher in female than in male mice, with steeper dose-dependent increases in blood levels seen in females. In a separate experiment, UM-HET3 mice at UT, at 26 months of age, were fasted overnight and then allowed access to food containing rapamycin at 42 ppm for 1 h. Blood samples were then taken at intervals for the next 6 h. The results, shown in Fig. S2 (Supporting information), right panel, show higher levels of rapamycin in blood of female mice at each time point tested. Although such differences might reflect differences in food consumption, we conclude that female UM-HET3 mice are likely to have higher blood levels of rapamycin than males, at any age, given equal doses of rapamycin in the chow. A similar pattern of sexual dimorphism was noted in studies of C57BL/6 mice and in a segregating (C57BL/6 × C3H/HeJ) stock used a control for studies of human alpha-synuclein (R. Strong and M. Javors, unpublished data).

Maximum likelihood calculations were conducted to estimate fits to the Gompertz parameterization for each of the rapamycin survival tables. Figure S3 (Supporting information) shows both smoothed and fitted results, for each sex, comparing control mice to those treated with the highest dose of rapamycin, and parameter estimates are shown in Table S2 (Supporting information). For the females at 42 ppm, the estimate for the Gompertz ‘a’ (intercept) parameter, indicative of underlying, age-independent mortality risk, is 20-fold lower in rapamycin mice compared with controls (*P* < 0.05). Females exposed to rapamycin at 4 and 14 ppm had intermediate values, not significantly different from controls. The estimate for the Gompertz ‘b’ (slope) parameter, an index of age-dependent change in mortality risk, is significantly higher in females receiving rapamycin at 42 ppm. The implications of this are uncertain, however, because in small data sets of this kind, early deaths are few and tend to produce elevation of the slope parameter in association with decreases in the intercept value. For males, as in females, the highest rapamycin dose led to a significantly lower ‘a’ parameter, but for males, there were no significant drug effects on the Gompertz slope. These estimates, for both sexes, should be interpreted with caution, because the calculations do not adjust for site-to-site variation, statistical power is low, and therefore, confidence intervals are correspondingly wide.

Our standard protocol calls for the removal of all mice in cages in which fighting has led to serious injuries; both dominant and nondominant, that is, wounded, mice are removed from the protocol to prevent over-representation of mice with specific personality characteristics. This policy typically leads to the removal of approximately 0–5% of the cages of male mice, and in the current cohort, only 1% of control male cages were culled because of fighting. We noted, however, that the proportion of male mice removed because of fighting was elevated in the group exposed to the highest dose of rapamycin, leading to the removal of 11% of the cages at TJL, 18% at UM, and 22% at UT. We do not know whether this higher incidence of serious fight wounds reflects changes in behavior of dominant or nondominant animals, or rapamycin-mediated interference with wound healing. A more detailed behavior analysis, with controlled tests on wound healing rates, would help to address this unexpected observation.

**Figure 2:**
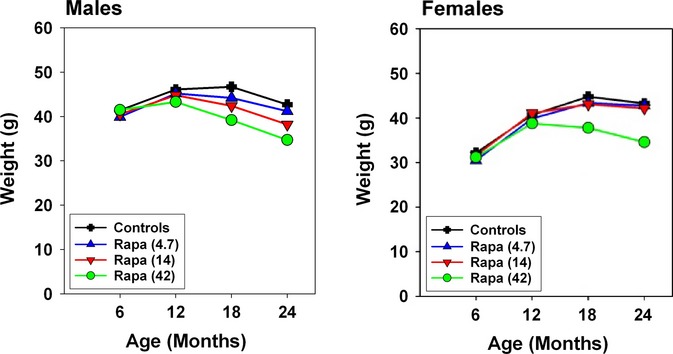
Weight changes in control and rapamycin-treated mice. Weights of control and rapamycin-treated mice at varying ages. Rapamycin treatment was initiated at 9 months of age. Points indicate mean values, pooled across the three test sites, for groups of approximately 150 mice in each rapamycin group and 300 control mice.

In principle, a drug might lead to longevity extension by making food unpalatable, thus reducing food intake sufficiently to induce true DR. Figure 2 shows weight at ages 6, 12, 18, and 24 months in rapamycin-treated and control mice. A similar pattern of rapamycin effects was seen at each of the three test sites (not shown), although weights of males and females at UM were, as in all previous ITP cohorts, consistently lower than weights of mice at the other two sites. The highest dose of rapamycin leads to 16–20% lower body weight (at 18 and 24 months, respectively) in female mice, but the other two doses reduce body weight by only 1–4%, making it seems unlikely that the lifespan effects represent caloric restriction per se. In males, weight is reduced by 11% and 19% at the two highest rapamycin doses. When UM-HET3 mice are exposed to lifelong dietary restriction, effects on weight and lifespan were similar in both sexes (Flurkey *et al*., 2010). An MRI method was used at the UT site to evaluate lean and fat mass in a small group of 9-month-old mice (*N* = 5–8 mice of each sex for each rapamycin dose). In males, the highest dose of rapamycin decreased the proportion of weight due to fat (29.3% vs. 35.8%, *P* = 0.0002 by *t*-test). A similar effect was seen in females (36.6% in treated mice vs. 43.4% in controls), but did not reach statistical significance (*P* = 0.13). The results are shown in Fig. S4 (Supporting information).

**Figure 3:**
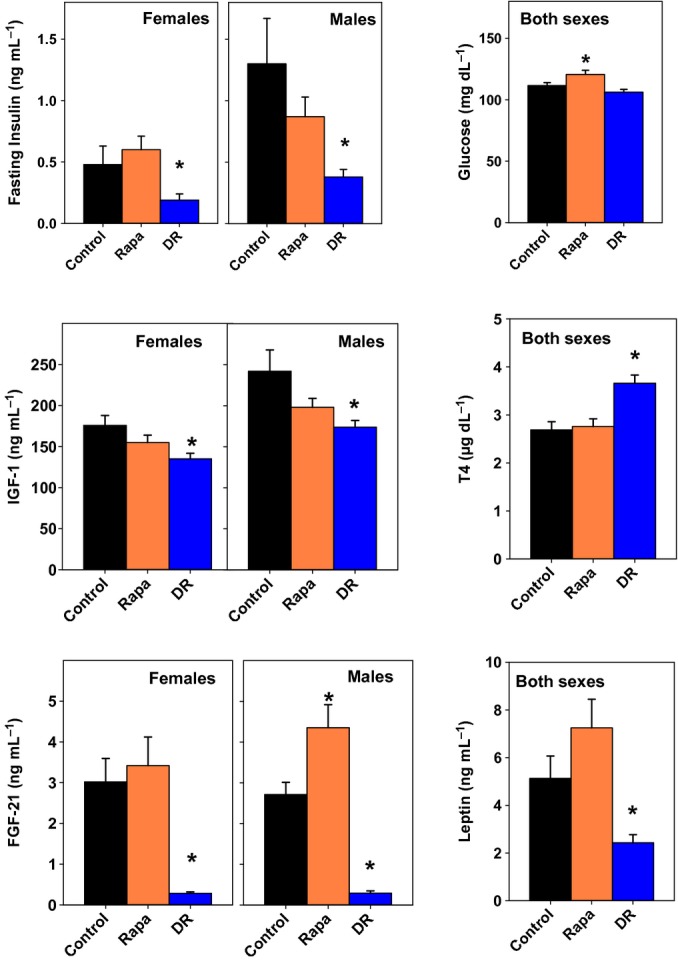
Metabolic status of dietary restriction (DR) and rapamycin-treated mice. Endocrine and metabolic endpoints in control, rapamycin-treated (at 14 ppm), and DR mice evaluated in plasma at 9 months of age after an overnight fast. DR and rapamycin exposure were begun at 4 months of age. Each bar shows mean and standard error of the mean for 8–10 animals of each sex. For insulin, IGF-1 and FGF-21, anova indicated a significant difference between males and females, and thus, each sex was analyzed separately. Sexes are pooled for the analyses of glucose, T4, and leptin. An asterisk indicates significant difference from control mice at *P* < 0.05. DR and Rapa mice differed at *P* < 0.006 for each endpoint, except IGF-1, for which there was no significant difference.

Figure 3 presents a series of endocrine and metabolic indices evaluated in UM-HET3 mice exposed to rapamycin at 4 months of age, at the middle dose of 14 ppm, in parallel to a cohort placed on a 40% DR diet at the same age. These mice were housed at UM and were not part of the longevity cohort. Fasting insulin levels (panel A) were significantly diminished by DR in both males and females; rapamycin did not lead to significant alterations in fasting insulin in either sex. Fasting glucose levels were significantly increased in the rapamycin-treated mice (panel B). Plasma IGF-1 was reduced by DR in both sexes; rapamycin showed a similar trend, but did not lead to a significant reduction in IGF-1 in either sex (panel C). Thyroid hormone T4 was increased by DR, but not by rapamycin (panel D). FGF-21, a hormone produced by the liver in response to acute starvation (35), was dramatically lower in plasma of the DR mice, consistent with observations in other stocks (A. Bartke, L. Y. Sun and A. Spong, unpublished data); rapamycin, in contrast, led to a significant elevation in FGF-21 in male mice and did not alter FGF-21 in females (panel E). DR mice had lower leptin levels, consistent with the known effects of DR on adiposity, but rapamycin did not cause a decline in leptin (panel F). In addition, testis weight, as a proportion of body weight in 12 month old males, is reduced by rapamycin (25% decline, *P* = 0.04), but not by DR (11% increase, *P* = 0.5). The DR and rapamycin mice differ significantly from one another (*P* < 0.004), for each of the endpoints shown in Fig. 3, except IGF-1. It is clear from this survey that the endocrine changes induced by 5 months of DR diet are in many ways distinct from those produced by 5 months of exposure to rapamycin at 14 ppm.

**Figure 4:**
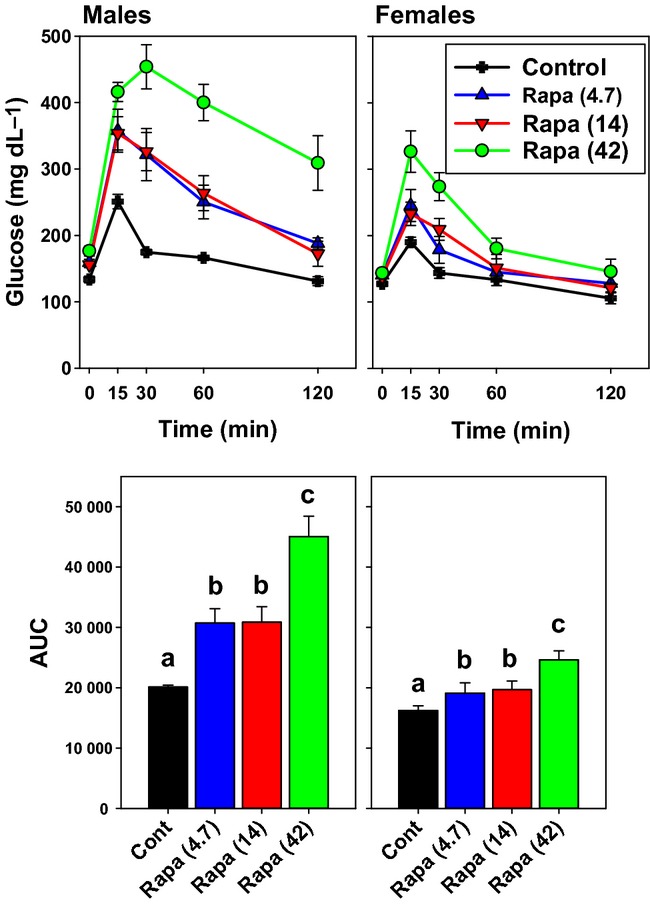
One-month exposure to rapamycin impairs glucose tolerance. UM-HET3 mice, at 4 months of age, were placed onto diet containing the indicated doses of rapamycin at UT and evaluated 1 month later for responses to intraperitoneal glucose injection. *N* = 5 of each treatment group for each sex. The upper panels show mean ± SEM for plasma glucose at the indicated times. The bottom panels show integrated area under the glucose curve as mean ± SEM; bars that share a letter code are not statistically significant by *t*-test.

A separate experiment conducted at the UT site evaluated glucose tolerance in UM-HET3 mice exposed to rapamycin for one month starting at 4 months of age. As shown in Fig. 4, glucose tolerance is impaired in both sexes even at the lowest dose of rapamycin, with further impairment seen in mice exposed to the highest rapamycin dose. In a two-factor analysis of variance, the effects of sex and rapamycin level were each significant at *P* < 0.0001, and there was a significant interaction term (*P* = 0.003), showing that the effects of rapamycin on glucose tolerance were more dramatic in males than in females. A parallel study of glucose responses after insulin injection (not shown) revealed that rapamycin-treated mice, in general, were mildly insulin resistant. Interestingly, we also noted a paradoxical increase in plasma glucose in males injected with insulin at 14 or 42 ppm, which was not seen in control males or in any of the female groups. It is possible that the increase in glucose seen after insulin injection into rapamycin-treated males reflects a response to the stress of injection, but additional work would be needed to address this idea.

We also evaluated hepatic expression of 52 mRNAs encoding enzymes involved in metabolism of xenobiotic chemicals and toxins (‘XME’, xenobiotic metabolizing enzymes). Genes encoding XMEs are frequently overexpressed in *Caenorhabditis elegans* mutants that show increased lifespan (Melo & Ruvkun, 2012) and have been postulated (Gems, 2007) to play a causal role in worm lifespan extension. Similarly, many XME mRNAs are elevated in liver of male Ames dwarf mice (Amador-Noguez *et al*., 2005), consistent with the idea that protection against the toxic effect of ingested or endogenously produced chemicals may play a role in the long life of this pituitary-deficient dwarf mouse. A survey of a subset of XME genes in male mice has also shown elevation of many of these mRNA in Snell dwarf and DR mice, as well as in rapamycin-treated mice (Steinbaugh *et al*., 2012). These previous reports, however, did not include data on female mice and focused on the set of XME genes that were known to be expressed at higher levels in female (control) mice than in males (Waxman & Holloway, 2009).

**Figure 5:**
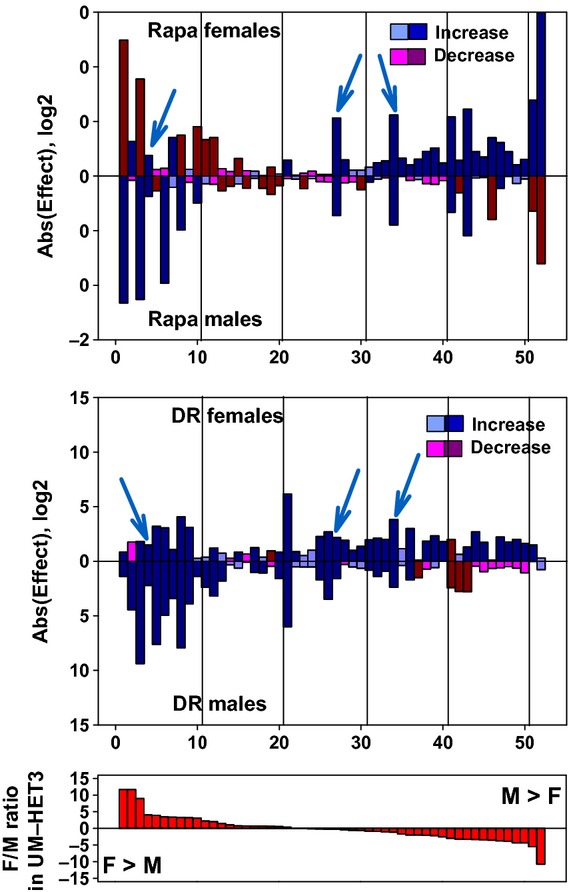
Expression of mRNA for liver genes involved in xenobiotic metabolism. Effects of rapamycin (top panel) and DR diet (middle panel) on hepatic mRNA levels for 52 genes related to xenobiotic metabolism. Top and middle panels: mice were placed on rapamycin (14 ppm) or DR diet at 4 months of age and euthanized at 12 months of age. There were six males and six females in the control group and in each of the two treatment groups. All mice were housed at UM. Data from treated female mice are shown in the top of each panel, bars pointing upwards, and data from treated male mice are shown in the bottom half, with bars pointing downwards. The length of each bar is shown on a log2 scale, as the ratio of treated mice divided by control mice. Bars shown in blue are increases compared with control, and bars shown in red are decreases compared with control mice. Thus, a blue bar with a value of eight represents an increase of 2^8^=256-fold above control levels, and a red bar with a value of eight represents a decline of 256-fold below control levels. Dark blue and dark red bars show genes for which the nominal (unadjusted) *P*-value is *P* < 0.05. Lighter blue and red bars shown genes that do not reach this arbitrary significance threshold. The first gene, for example, Sult2a2 (see Table S3), is increased by DR, slightly but significantly, in both males and females, is dramatically increased (by 2^11.6^ = 3100-fold) by rapamycin in males, but shows a large (2^12.4^ = 5400-fold), significant decline in rapamycin-treated females. The cyan arrowheads point out the three mRNA that are elevated by both DR and rapamycin in both sexes.The bottom panel shows female/male ratios, for untreated UM-HET3 controls; genes at the left are expressed at levels approximately 2^12^-fold higher in female controls, and genes at the right are expressed roughly 2^11^-fold higher in male controls.

In the current study, we evaluated expression of XME mRNAs in liver of DR, rapamycin-treated (14 ppm), and control mice at 12 months of age, using a set of mRNAs that varied widely in the degree and direction of sexual dimorphism. The gene expression data are shown in Table S3 (Supporting information) and represented graphically in Fig. 5. Panel A of Fig. 5 compares rapamycin to control mice, and Panel B compares the same genes in DR versus control mice. In each figure, genes that are preferentially expressed in control females are shown at the left, and genes preferentially expressed in control males are shown at the right. Bars shown in blue indicate mRNA whose expression is increased by rapamycin (Fig. 5A) or by DR (Fig. 5B), and bars shown in red represent genes that decrease in the treated mice compared with controls. The extent of the alteration is shown on a log2 scale; the largest effects therefore exceed 2^10^, or 1000-fold.

The effects of the two interventions on XME gene expression are clearly quite distinct. There is a tendency for ‘female-dominant’ XME genes to be diminished by rapamycin in females and increased by rapamycin in males. ‘Male-dominant’ XME genes show the opposite pattern, tending to increase in females treated with rapamycin and to decrease in rapamycin-treated males. For these sexually dimorphic mRNA, then, rapamycin tends to diminish sexual disparities in both males and females. There are, however, many XME that do not follow this pattern, and large, significant effects are also seen among mRNAs that show little or no sexual dimorphism. Only six of the 52 mRNAs show (nominally) significant increases in both males and females exposed to rapamycin, and only a single mRNA shows a significant decrease in both sexes.

The pattern of XME expression in livers of DR mice bears some resemblance to that seen after rapamycin, in that there is a tendency for female-dominant genes to be increased in male mice and for male-dominant genes to be increased in female mice after DR. Twenty of the 52 tested mRNA show significant increases in both sexes in response to the DR diet, and one shows a significant decrease. Of these 21 DR-modified XME mRNAs, only three show parallel changes in males and in females exposed to rapamycin. These three are Cyp3a41, Aldh3a2, and Gstt1 at positions 4, 27, and 34 on the plots. Additional work would be needed to see whether levels of the corresponding proteins did or did not show parallel changes. This survey, although limited, does not support the hypothesis that elevation of mRNA for hepatic XME is a feature of rapamycin exposure, or the idea that gene expression changes induced by rapamycin closely parallel those seen after dietary restriction.

## Discussion

Rapamycin at 42 ppm leads to 26% increase in median lifespan in females and 23% in males, in each case larger than that seen in previous or current cohorts exposed to lower doses. Survival of the longest-lived 10% of the cohort was increased by 11% in females and by 8% in males, and the drug benefited male and female mice at each of the test sites. It is possible that larger lifespan effects might be seen at still higher doses of rapamycin, or in mice treated with rapamycin in combination with one or more agents that work through other pathways. The lifespan benefit is not accompanied, in either sex, by weight loss to the degree typical of mice exposed to DR (Flurkey *et al*., 2010), and the two lower doses increased female lifespan significantly without any apparent effect on weight. The middle dose of rapamycin (14 ppm) is the same as that used for an earlier, independent cohort (Miller *et al*., 2011). In the previous paper, the change in median lifespan for females was 18% and for males was 10%, averaged across the three test sites. In the current data set, the average increase for females was also 18% and for males was 5%, in reasonably good agreement with the initial report.

Rapamycin leads to a larger percentage increase in female longevity than in males, even though female controls live longer than male controls at TJL and UT. It seems likely that this differential effect, seen now in three consecutive independent cohorts, reflects higher levels of rapamycin in blood of female UM-HET3 mice compared with males given equal amounts of rapamycin in food. Our data show higher levels in young females at each of three rapamycin doses and higher levels in old females at varying times after consumption of rapamycin-containing food. Similar sex effects were noted in at least two other strains of mice (M. Javors and R. Strong, unpublished data). But it is also likely that males and females may differ in other ways in their response to rapamycin. For one example, gene expression changes induced by rapamycin in males differ radically from those produced in females, at least with respect to XME genes evaluated in Fig. 5. Hypotheses related to sex hormones, sexual dimorphism in immune tone, differences between males and females in GH pulse height (Waxman & Holloway, 2009), and sex-specific differences in fat distribution can be evaluated in future studies of rapamycin effect. Mutations that diminish GH production (Brown-Borg *et al*., 1996) or activity of mTOR (Selman *et al*., 2009) can in some cases produce stronger effects in females than in mice, but this pattern is by no means universal (Ladiges *et al*., 2009). Two other agents tested by the ITP, specifically nordihydroguaiaretic acid (NDGA) (Strong *et al*., 2008) and acarbose (Harrison *et al*., 2013), also show sex-specific effects on lifespan, but these two agents affect lifespan in males preferentially. In addition, there is some evidence that the nonfeminizing estrogen 17-α-estradiol may also augment lifespan preferentially in male mice (Harrison *et al*., in press).

It seems likely that rapamycin-mediated increases in lifespan reflect both broad spectrum antitumor effects as well as deceleration of aging processes more generally. Terminal (end of life) necropsies conducted on mice exposed to 14 ppm rapamycin from nine months of age suggested that the spectrum of specific lethal illnesses, mostly neoplastic, was not altered by rapamycin, even though the age at death was higher in the treated mice compared with controls, suggesting a drug-induced postponement of tumor induction, progression, or lethal effect (Miller *et al*., 2011). Data from cross-sectional histopathology have also been published for mice at each of the three rapamycin doses tested here, euthanized at 22 months of age (Wilkinson *et al*., 2012), and showed evidence for rapamycin-dependent improvement in age-associated changes in liver, myocardial, endometrium, adrenal, and tendon, in addition to suggestive (but not statistically significant) effects in ovary, thyroid, and lung. Rapamycin also retarded age-dependent declines in spontaneous in-cage activity (Miller *et al*., 2011; Wilkinson *et al*., 2012). Brief treatment of 22-month-old C57BL/6 mice has been found to improve turnover of hematopoietic stem cells and B-cell production in the bone marrow and to improve responses to influenza vaccination, sufficient to protect aged mice from lethal viral infection (Chen *et al*., 2009). Rapamycin administered to mice prone to Alzheimer’s-like brain pathology delays development of tau and amyloid-dependent pathology, leads to improvements in cognitive function (Caccamo *et al*., 2010). In addition, rapamycin can reverse age-dependent defects in cardiac function (Flynn *et al*., 2013), and can protect against atherosclerotic change in mice prone to such disease (Mueller *et al*., 2008). These results argue that rapamycin can slow the effects of aging on many cell and tissue types and suggest that postponement of lethal neoplasia could be mediated by direct effects both on the tumor cells and on age-sensitive antitumor defenses. There are, however, age-dependent changes in male C57BL/6 mice which are not prevented by rapamycin, and some of the protective effects of this drug appear to reflect immediate benefits rather than delay in age-dependent change (Neff *et al*., 2013).

The documented decline in TOR activity seen in DR rodents (Sun *et al*., 2009) supports the idea (Kaeberlein & Kennedy, 2009) that lower TOR function may be responsible for lifespan extension in both kinds of mice. Our data do not disprove this idea, but they do document many differences between DR and rapamycin effects on characteristics postulated to play a role in age-dependent pathophysiology (Figs 3 and 4). Rapamycin does not lead to the decline in fasting insulin, leptin, IGF-1, or FGF-21 or to the increase in T4 seen in UM-HET3 mice after 5 months of DR, but does cause an increase in fasting glucose not seen in the DR animals. At this point, it is not known which changes, in which cell(s), lead to slower aging in either DR or rapamycin-treated mice, so it is not possible to know whether the two interventions alter aging through shared mechanisms. We believe that our data do show, however, that the effects of rapamycin, at the doses used, differ in some ways from those produced by DR, making it less likely that the lifespan effect is due simply to diminished calorie or nutrient intake. A detailed tabulation of the effects produced by rapamycin, various forms of dietary restriction, other drugs that extend lifespan (Harrison *et al*., 2013), and life-extending mutants may help direct experimental attention to specific shared pathways.

There is now good evidence that rapamycin can alter glucose metabolism after various times and levels of exposure in mice. For example, in both young and old UM-HET3 mice, rapamycin treatment at 14 ppm for 3 weeks causes glucose intolerance and increased gluconeogenesis (Lamming *et al*., 2013). Chronic injection of rapamycin also causes hepatic insulin resistance in young C57BL/6 mice and Sprague-Dawley rats (Houde *et al*., 2010; Lamming *et al*., 2012). There is also evidence that rapamycin is toxic to pancreatic β-cells both *in vitro* and *in vivo* (Barlow *et al*., 2013). As increased insulin sensitivity is a notable feature of longevity extension in pituitary dwarf mutants (Dominici *et al*., 2002) and in DR rodents (Barzilai *et al*., 1998; Fok *et al*., 2013), the deficit in glucose homeostasis consistently seen in rapamycin-treated mice is unexpected and suggests that the lifespan benefit of this drug might be extended still further by combination of rapamycin with an insulin sensitizing agent like metformin. On the other hand, it is interesting to note that we found the dose of rapamycin that extends lifespan to the greatest degree (42 ppm) is also associated with the largest deficits in glucose metabolism. The data in Fig. 4 indicate that the loss of glucose tolerance is seen as early as 1 month after the start of rapamycin treatment and is dose dependent. The rapamycin effect is seen in both sexes, but is more dramatic in male mice, at least at this time point. It is possible, although clearly speculative, that the more dramatic loss of glucose control seen in males could contribute to the lower lifespan effect produced by rapamycin in males. Males and females also differ in that only in males does rapamycin produce a paradoxical hyperglycemia after insulin injection (data not shown), perhaps reflecting male-specific sensitivity to handling stress or insulin-mediated stress.

The association between insulin sensitivity and longevity is still a topic of active investigation. One recent study, for example, found that increasing insulin sensitivity can lead to shorter lifespan in mice, and the mice with reduced insulin sensitivity did not necessarily have shorter lifespan (Nelson *et al*., 2012). The ITP group has initiated an experiment to see whether the lifespan effect of rapamycin on UM-HET3 mice is altered, in either direction, by administration of metformin.

In addition, the effects of the two interventions on hepatic expression of XME genes show little overlap in pattern or detail (Fig. 5). Our initial working hypothesis, based on the prior observations and speculation (Gems, 2007), was that many XME genes would be elevated in both males and females of the DR and rapamycin groups. In addition, the idea that TOR inhibition is a key feature of the DR longevity effect implied that at least some subset of XME genes would be changed in parallel in both treatment groups, and for both males and females. Neither of these ideas receives much support from the data in Fig. 5. Only three of the 52 genes tested showed an elevation in both sexes of both treatment groups. Sex-specific differences in the amplitude of pulses in serum growth hormone levels have been shown (Waxman & Holloway, 2009) to exert a major influence on hepatic expression of XME genes. Male mice typically have higher peak levels and lower trough levels of GH than females, leading to repression in males of many XME genes that are strongly expressed in females. Studies of male Ames (Amador-Noguez *et al*., 2005) and Snell (Steinbaugh *et al*., 2012) dwarf mice have shown strong and consistent activation of XME genes, but focused largely on genes that are expressed more highly in female controls; in retrospect, these findings are plausibly interpreted as the loss of repression of female-specific XME genes when GH pulses are obliterated in the male pituitary mutants. Of the 10 mRNA with the highest ratio of female/male expression in controls (see bottom panel of Fig. 5), all 10 are increased by DR in males, as are seven of the ten in rapamycin-treated males. In principle, this effect might indicate a loss of the GH-dependent repression of XME expression in males. The observations in female mice, however, complicate the story, in that many of these 10 genes are also elevated in DR females, and six show a decline in females exposed to rapamycin. Both DR and rapamycin tend to increase expression of male-dominant genes in female mice and to decrease expression of these same genes in male mice, but it is not clear how such neutralization of the sexual dimorphism in XME expression might contribute to the extension of both male and female lifespan seen in rapamycin-treated and DR-treated (Flurkey *et al*., 2010) UM-HET3 mice. Data on expression of XME genes in other models of delayed aging in mice may provide a better understanding of the role, if any, of XME gene expression in the control of mouse lifespan, but the current data give few hints of common features, shared by the two interventions in both sexes, which might contribute to the delay in aging and late-life diseases.

Rapamycin, like DR, slows many aspects of aging in UM-HET3 mice (Wilkinson *et al*., 2012), delays cancer (Livi *et al*., 2013), and extends lifespan. While we do not know how either intervention produces these beneficial effects, it will be of great interest to learn whether rapamycin and DR share one or more critical causal mechanisms or act through different pathways. The growing availability of drugs, diets (Orentreich *et al*., 1993), developmental interventions (Sun *et al*., 2009), and mutations (Brown-Borg *et al*., 1996; Ladiges *et al*., 2009) that extend mouse mean and maximal lifespan provides a battery of model systems in which to look for pathways that may be shared among these different anti-aging interventions.

## Experimental procedures

### Mice, husbandry, and survival data

Details of breeding and husbandry, including preparation of encapsulated (enteric-released) rapamycin, have been described at length in earlier papers (Miller *et al*., 2007, 2011; Strong *et al*., 2013), as have results of both end of life (Miller *et al*., 2011) and midlife (Wilkinson *et al*., 2012) necropsy analyses. In brief, genetically heterogeneous UM-HET3 mice were produced by a cross between CByB6F1/J mothers (JAX #100009) and C3D2F1/J fathers (JAX #100004), at each of three test sites: the Jackson Laboratory (TJL), the University of Michigan (UM), or the University of Texas Health Science Center at San Antonio (UT). They were housed at three male mice or four female mice per cage from weaning and at 9 months of age were given a diet containing encapsulated rapamycin at 4.7, 14, or 42 ppm (mg of drug per kg of food). Mice that died were not replaced, so cage density declined at older ages. Cages were inspected daily. Date of death was noted for mice found dead, and mice found to be so ill that they were expected to die within the next 24–48 h were euthanized, with the date of euthanasia taken as the date of death for life table calculations. At the time of analysis, 532 control mice had died, and two control mice (0.4%) were alive; 749 mice exposed to rapamycin had died, and 15 (1.9%) were alive. In addition, five mice were removed prior to natural death because of experimental accidents (for example, death during implantation of ID tag); six were removed for humane reasons because of dermatitis; 74 males were removed according to our protocol policy which requires euthanasia of all mice in cages in which any mouse is found to have extensive fight wounds (Miller *et al*., 2007); and 104 mice, all at TJL, were removed for use in a separate study of immune function.

### Assessment of glucose and hormones in plasma

Mice at UM were fasted overnight (18 h), after which blood samples for hormone measurements were collected via tail nick, without anesthesia, using heparin as an anticoagulant. Samples were obtained between 8 and 10 am. Glucose levels were determined using a handheld glucometer, and the plasma samples then stored at −80 °C prior to the assessment of hormone concentrations. Plasma IGF-1 levels were determined with a double-antibody RIA kit (Cat. # IGF-21, ALPCO, Salem, NH, USA). For the IGF-1 RIA, the assay was run at one-quarter of the manufacturer’s recommended protocol volumes. Plasma T_4_ levels were quantified using a monoclonal solid-phase RIA kit (Cat. # 06B-254011, MP Biomedicals, Orangeburg, NY, USA). For the T_4_ RIA, the assay was run at 40% of the volumes stated in the manufacturer’s protocol. Insulin levels were quantified via a two-site enzyme immunoassay kit (Cat. # 80-INSMSU-E01, ALPCO). Plasma leptin levels were determined using an ELISA sandwich assay (Cat. # 90030, Crystal Chem Inc., Downers Grove, IL, USA). Leptin samples were performed in two batches, and a correction factor used to adjust the mean level in each batch to the same level prior to further analysis. Plasma FGF-21 levels were quantified using an ELISA sandwich assay. (Cat.# EZRMFGF21-26K, Millipore, St. Charles, MO, USA). All samples were assayed in duplicate.

### Glucose tolerance

UM-HET3 mice housed at UT were fed control, 4.7, 14, and 42 ppm rapamycin diets starting at 4 months of age for 1 month. For the glucose tolerance tests, mice were fasted 6 h (09:00–15:00) prior to each test and then injected intraperitoneally with glucose (1.5 g kg^−1^) in saline. Blood glucose levels were measured at indicated time points from tail vein bleeding by handheld glucometer (LifeScan, Milpitas CA, USA). Area under curve (AUC) was calculated for each animal using the trapezoid method.

### Gene expression measurements

Liver was taken from mice at 12 months of age, after 8 months exposure to control, rapamycin, or DR diets. Total RNA was isolated from mouse livers using Trizol (Invitrogen, Carlsbad, CA, USA), cleaned using the Qiagen RNeasy mini RNA cleanup protocol (Qiagen, Valencia, CA, USA), and quantified using a Nanodrop ND-100. Total RNA was then transcribed to cDNA using iScriptTMcDNA reverse transcription kits (1708891; Bio-Rad, Hercules, CA, USA). Reverse transcription products were amplified using Power SYBR Green PCR Master Mix (Applied Biosystems, Foster City, CA, USA) and PCR primers (Integrated DNA Technologies, Coralville, IA, USA). RT–PCR was performed using abi prism sequence detection system instrument and software (Applied Biosystems). Ct data, indicating the number of amplification cycles needed to achieve threshold, were available from six male and six female mice per treatment group. The effect of rapamycin or DR diet was estimated as the difference between Ct for the mutant mouse and Ct for sex-matched littermate controls, with a positive value in cases where expression of the mRNA was increased by diet compared with control animals. Because a change in one unit for Ct reflects a twofold change in mRNA abundance, effect sizes in the figures and tables reflect powers of 2. Thus, an effect size of 5 indicates an increase in mRNA concentration of 2^5^ = 32-fold.

### Determination of rapamycin levels in plasma by tandem HPLC-MS

This method has been described elsewhere (Livi *et al*., 2013).

### Body composition

Fat and lean mass were evaluated by quantitative magnetic resonance imaging (QMR) with the EchoMRI-3-in-1 system (Echo Medical System, Houston, TX, USA), as described (Tinsley *et al*., 2004). The procedure requires immobilizing the mice in plastic restrainer tubes without sedation and takes <2 min per mouse.

### Statistics

Survival curves were compared using the log-rank test, with stratification by site for comparisons that pooled data across sites. Mice that had been removed from the protocol, for example, because of an accident or fighting, were treated as alive on the day of removal and lost to follow up thereafter. Statements about maximal lifespan were based on the method of (Wang *et al*., 2004), using Fisher’s exact test at the age at which 90% of the mice in the pooled survival table had died. Point estimates of mortality rates (*u*_*x*_) were obtained by pooling deaths and censored observations into 2-week census intervals and calculating *u*_*x*_ *= −ln(p*_*x*_*)*, where *p*_*x*_ is the probably of surviving through interval age *x*. Parameter estimates for the Gompertz mortality model were obtained using maximum likelihood analysis of actual (i.e., not pooled) observation times (Pletcher, 1999). For metabolic tests, two-factor anova (sex, treatment) was used to determine whether there was a significant difference between males and females; if a sex effect was noted, then the data were analyzed separately for each sex, and the data were pooled for analysis if no sex effect was noted. Differences between treated and control groups, or in some cases between DR and rapamycin groups, were evaluated by *t*-test, and nominal *P*-values were reported without adjustment for multiple comparisons. For the gene expression studies, unadjusted Ct values (which are on a log2 scale) were compared for each gene between control and treated mice, using a *t*-test with no adjustments for multiple comparisons. Error bars shown in the figures indicate standard errors of the mean, and all *P*-values represent two-sided tests. Table S4 (Supporting information) provides information on sensitivity and reproducibility of the hormone tests shown in Fig. 3.

## Supporting Information

**Fig. S1** Survival curves at each of the three test sites. **Fig. S2** Sexual dimorphism in rapamycin blood concentrations. **Fig. S3** Gompertz plots for control and rapamycin-treated (42 ppm) mice. **Fig. S4** Fat mass, as a proportion of total mass, in 9-month-old mice exposed to various doses of rapamycin from 4 months of age. **Table S1** Survival statistics for each test site. **Table S2** Estimation of Gompertz parameters for rapamycin-treated mice. **Table S3** Effects of DR and Rapamycin on expression levels in liver of 52 mRNA for XME genes. **Table S4** Characteristics of the assays used for the work shown in Fig. 3.
