# Supplementary material for: Rapamycin-mediated lifespan increase in mice is dose and sex dependent and metabolically distinct from dietary restriction
Source: Aging Cell. 2014 Feb 9;13(3):468–77. doi: 10.1111/acel.12194 (PMC4032600; doi:10.1111/acel.12194)
Supplement: Supplementary file 1 — Fig. S1 Survival curves at each of the three test sites. Fig. S2 Sexual dimorphism in rapamycin blood concentrations. Fig. S3 Gompertz plots for control and rapamycin-treated (42 ppm) mice. Fig. S4 Fat mass, as a proportion of total mass, in 9-month-old mice exposed to various doses of rapamycin from 4 months of age. Table S1 Survival statistics for each test site. Table S2 Estimation of Gompertz parameters for rapamycin-treated mice. Table S3 Effects of DR and Rapamycin on expression levels in liver of 52 mRNA for XME genes. Table S4 Characteristics of the assays used for the work shown in Fig. 3. [file acel0013-0468-sd1.docx]

**Supplemental Figure S1: Survival curves at each of the three test sites.**

| 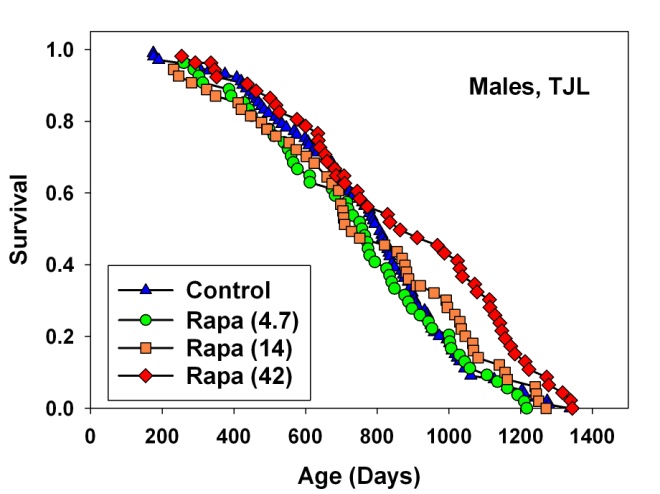 | 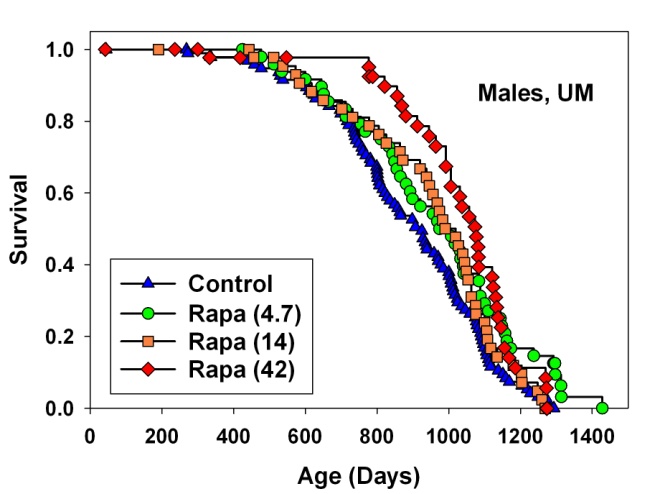 | 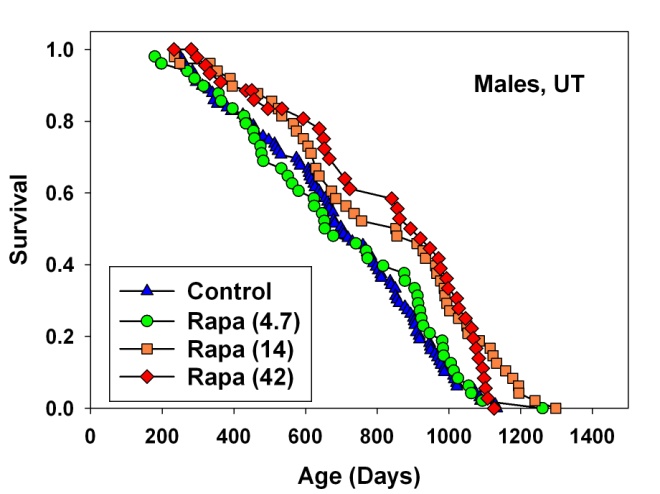 |
| --- | --- | --- |
| 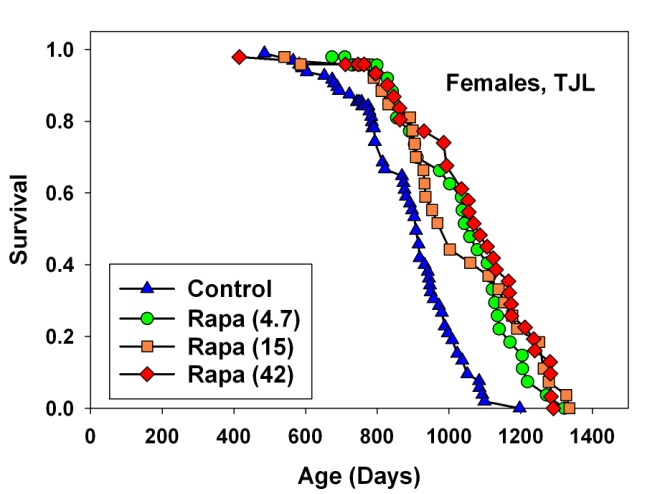 | 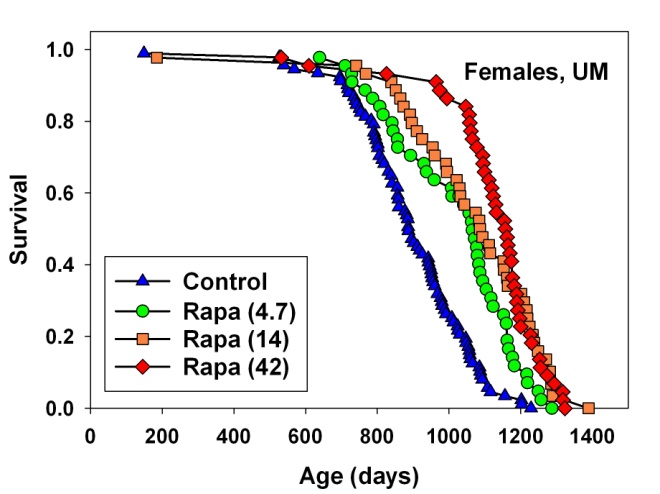 | 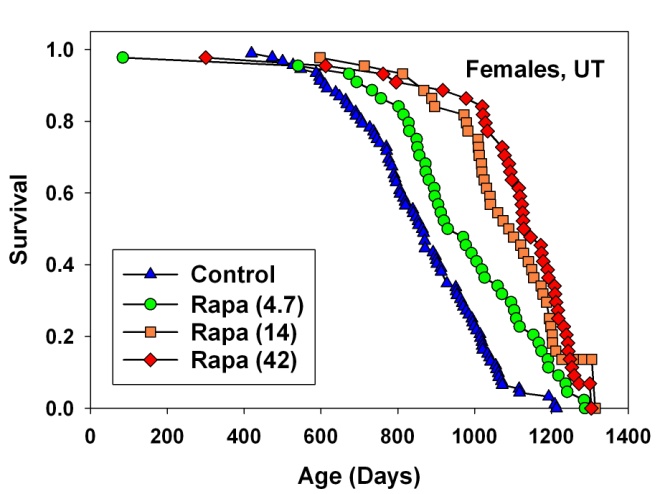 |

**Supplemental Figure S2: Sexual Dimorphism in Rapamycin Blood Concentrations**

| **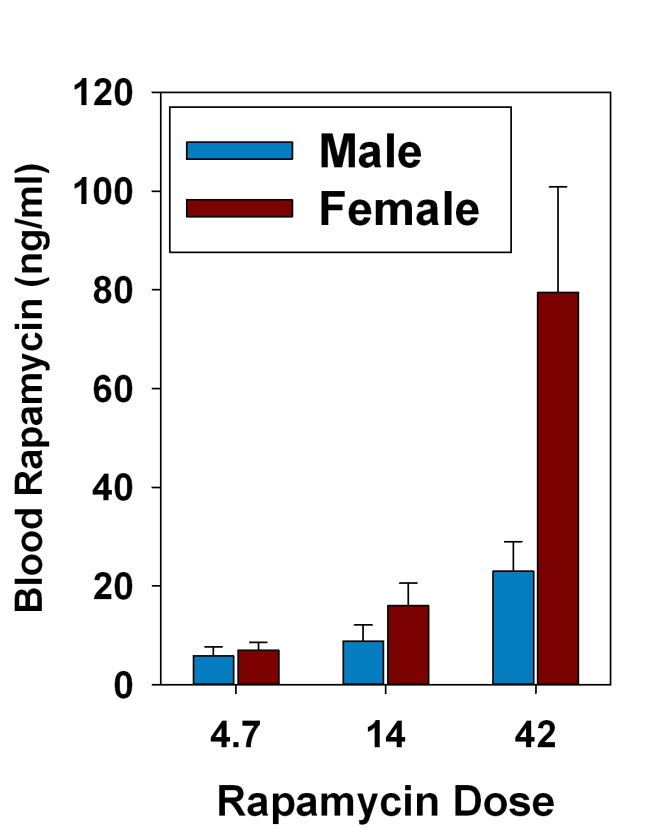** | **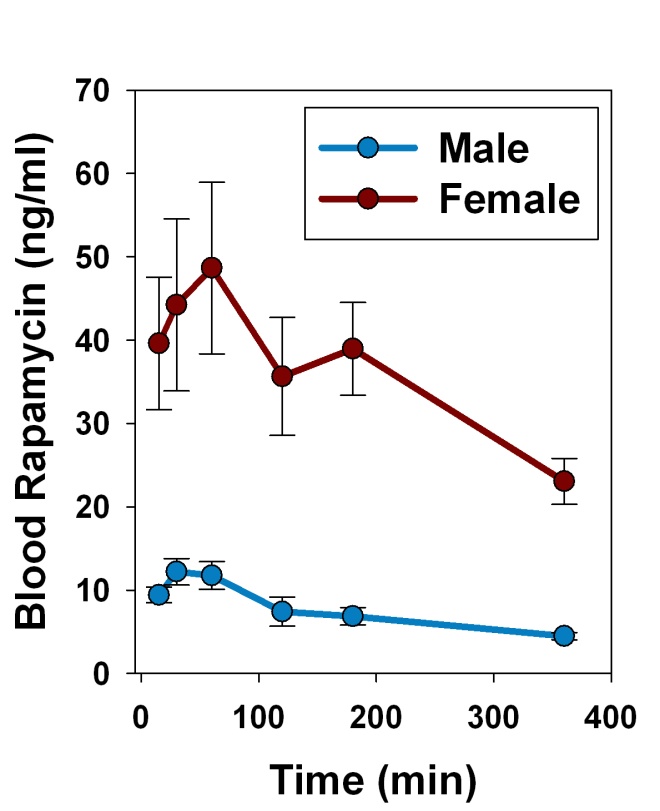** |
| --- | --- |

Legend for Supplemental Figure S2. Left panel: UM-HET3 mice at UT, age 3 – 4 months, were placed on diets containing rapamycin at the indicated dose for 5 months. Blood samples were then evaluated for rapamycin content. Bars indicate mean ± SEM for N = 4 to 8 mice of each sex. ANOVA documented significant effects of dose and sex, and a significant interaction term indicating that the effect of chow level on blood level differed between the two sexes. Right panel: UM-HET3 mice at UT, age 26 months, were fasted overnight and then allowed access to chow containing rapamycin at 42 ppm for one hour. Chow was removed, and blood rapamycin evaluated at intervals from 15 to 360 minutes. Symbols show mean ± SEM for N = 5 mice of each sex.

**Supplemental Figure S3: Gompertz plots for Control and Rapamycin-treated (42 ppm) mice**

| **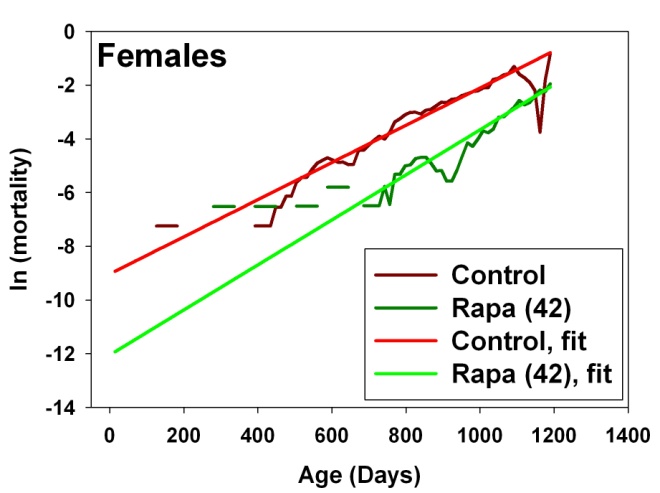** | **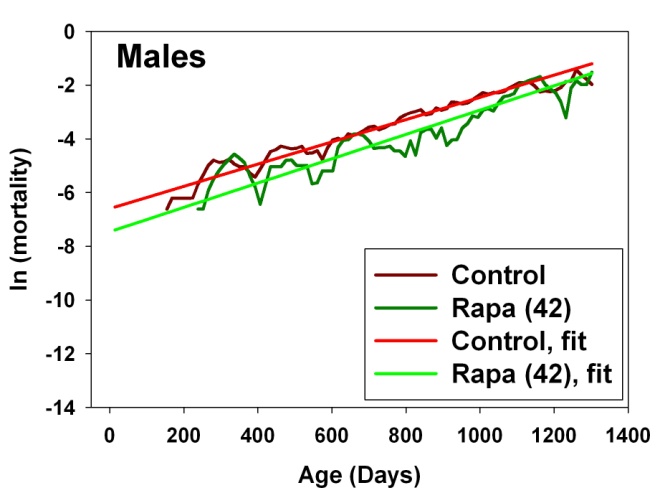** |
| --- | --- |

Legend for Supplemental Figure S3. Each panel shows the natural logarithm of mortality risk as a function of age for female (left) or male (right) mice, pooled across all three test sites, for control mice and mice treated with rapamycin at 42 ppm. Straight lines show the best fit of the data to the Gompertz model, and bent lines show the actual data, smoothed over 10 week windows. Interruptions in the lines indicate windows in which no mice died. Mice exposed to rapamycin at 4 or 14 ppm produced intermediate results (not shown in the graphic.) Parameter estimates for all groups are shown in **Supplemental Table 2**.

**Supplemental Figure S4. Fat mass, as a proportion of total mass, in 9 month old mice exposed to various doses of rapamycin from 4 months of age.**


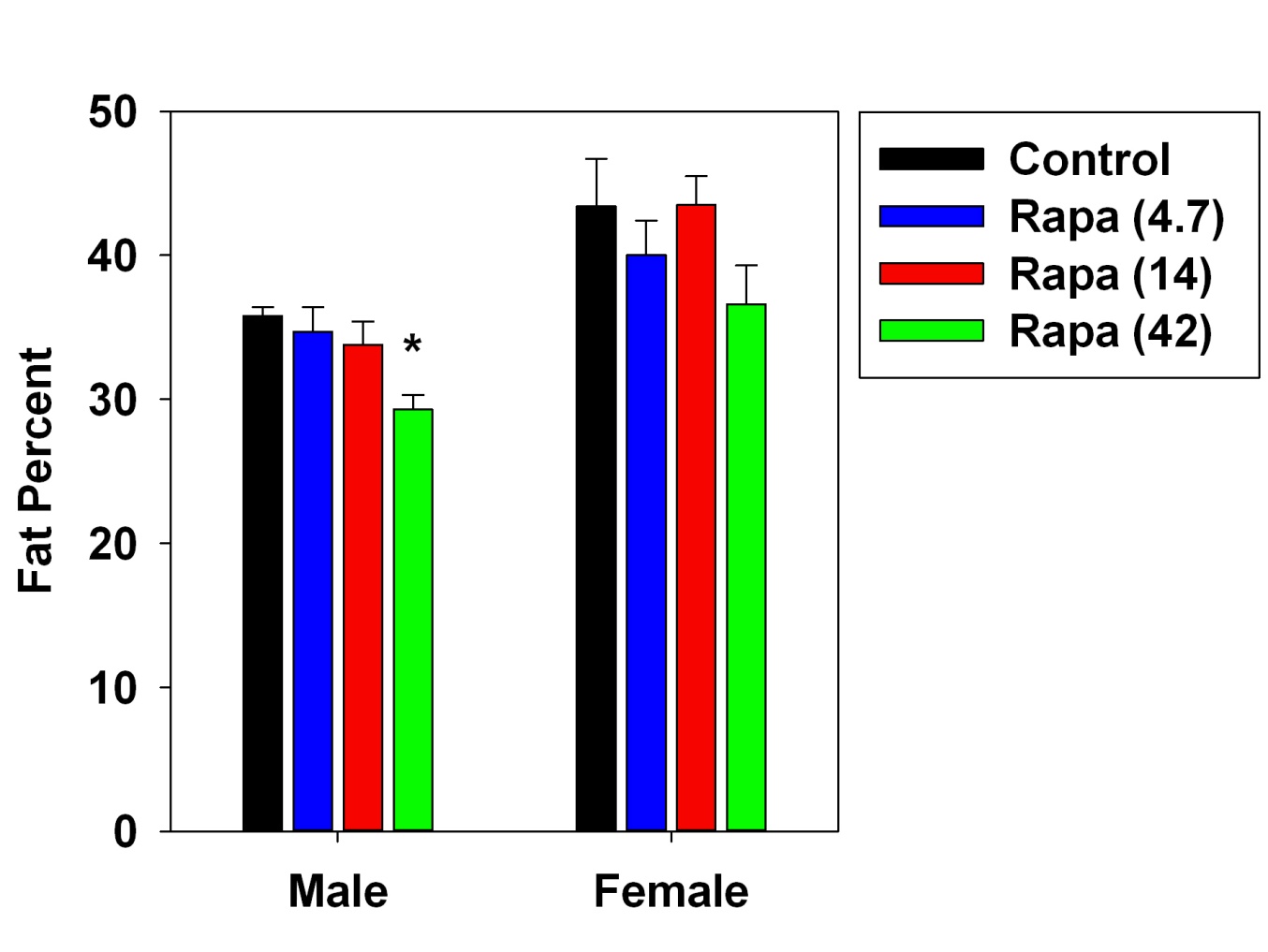


Legend for Supplemental Figure S4. Each bar represents mean and standard error for 5 to 8 mice. (*) indicates significant difference from control males (p = 0.0002 by t-test).

**Supplemental Table 1: Survival Statistics for Each Test Site**

| **TJL MALES** | **Median** | **Median Increase** | **Log-rank p-value** | **N** | **90th %ile age** | **90th %ile increase** | **Wang/**  **Allison** |
| --- | --- | --- | --- | --- | --- | --- | --- |
| Controls | 807 |  |  | 102 | 1061 |  |  |
| Rapa (4.7) | 757 | -6 | 0.6 | 54 | 1106 | 4 | 0.78 |
| Rapa (14) | 728 | -10 | 0.5 | 54 | 1163 | 10 | 0.39 |
| Rapa (42) | 864 | 7 | 0.014 | 54 | 1272 | 20 | 0.07 |
|  |  |  |  |  |  |  |  |
| **UM Males** |  |  |  |  |  |  |  |
| Controls | 925 |  |  | 99 | 1151 |  |  |
| Rapa (4.7) | 1077 | 16 | 0.02 | 51 | 1297 | 13 | 0.12 |
| Rapa (14) | 974 | 5 | 0.19 | 51 | 1203 | 5 | 0.22 |
| Rapa (42) | 1019 | 10 | 0.002 | 51 | 1270 | 10 | 0.48 |
|  |  |  |  |  |  |  |  |
| **UT Males** |  |  |  |  |  |  |  |
| Controls | 704 |  |  | 99 | 1013 |  |  |
| Rapa (4.7) | 677 | -4 | 0.55 | 51 | 1025 | 1 | 0.77 |
| Rapa (14) | 855 | 21 | 0.0005 | 51 | 1178 | 16 | 0.002 |
| Rapa (42) | 920 | 31 | 0.003 | 51 | 1098 | 8 | 0.04 |
|  |  |  |  |  |  |  |  |
| **TJL FEMALES** | **Median** | **Median Increase** | **Log-rank p-value** | **N** | **90th %ile age** | **90th %ile increase** | **Wang/**  **Allison** |
| Controls | 918 |  |  | 96 | 1051 |  |  |
| Rapa (4.7) | 1058 | 15 | < 0.0001 | 48 | 1219 | 16 | 0.001 |
| Rapa (14) | 1002 | 9 | 0.0001 | 48 | 1278 | 22 | < 0.0001 |
| Rapa (42) | 1106 | 20 | < 0.0001 | 48 | 1283 | 22 | < 0.0001 |
|  |  |  |  |  |  |  |  |
| **UM FEMALES** |  |  |  |  |  |  |  |
| Controls | 887 |  |  | 92 | 1089 |  |  |
| Rapa (4.7) | 1157 | 30 | > 0.0001 | 44 | 1217 | 12 | < 0.0001 |
| Rapa (14) | 1065 | 20 | > 0.0001 | 44 | 1285 | 18 | < 0.0001 |
| Rapa (42) | 1086 | 22 | > 0.0001 | 44 | 1274 | 17 | < 0.0001 |
|  |  |  |  |  |  |  |  |
| **UT FEMALES** |  |  |  |  |  |  |  |
| Controls | 864 |  |  | 92 | 1062 |  |  |
| Rapa (4.7) | 930 | 8 | 0.0008 | 44 | 1218 | 15 | 0.005 |
| Rapa (14) | 1089 | 26 | > 0.0001 | 44 | 1314 | 24 | 0.002 |
| Rapa (42) | 1128 | 31 | > 0.0001 | 44 | 1259 | 19 | < 0.0001 |

**Supplemental Table 2: Estimation of Gompertz parameters for rapamycin treated mice.**

| **Group** | **a (x 10^-5^)** | **b** |
| --- | --- | --- |
| Control females | 12.0 (7.0,22.0)^1^ | 0.097 (0.088,0.107)^1^ |
| Females: Rapa (4.7) | 6.0 (2.0,15.0)^1^ | 0.095 (0.082,0.109)^1^ |
| Females: Rapa (14) | 5.0 (2.0,14.0)^1^ | 0.090 (0.078,0.105)^1^ |
| Females: Rapa (42) | 0.59 (0.15,2.0)^2^ | 0.120 (0.101,0.136)^2^ |
|  |  |  |
| Control males | 1.4 (1.1,2.0)^2^ | 0.060 (0.052,0.064)^1^ |
| Males: Rapa (4.7) | 1.9 (1.2,3.1)^2,3^ | 0.049 (0.042,0.057)^1^ |
| Males: Rapa (14) | 1.1 (0.61,1.8)^1,2^ | 0.058 (0.050,0.067)^1^ |
| Males: Rapa (42) | 0.58 (0.30,1.1)^1^ | 0.063 (0.054,0.074)^1^ |

Maximum likelihood parameter estimates (with 95% confidence intervals) associated with the Gompertz model of mortality risk at age x (u_x_=ae^bx^) for cohorts of mice fed different doses of rapamycin and their corresponding vehicle-fed control cohort. Parameters with the same superscript(s) are statistically indistinguishable at α=0.05.

**Supplemental Table 3: Effects of DR and Rapamycin on expression levels in liver of 52 mRNA for XME genes.**

|  |  |  | Dietary Restriction | | | | Rapamycin | | | |
| --- | --- | --- | --- | --- | --- | --- | --- | --- | --- | --- |
|  |  |  | Females | | Males | | Females | | Males | |
| mRNA | Sex Ratio | Gene # | Effect | p-value | Effect | p-value | Effect | p-value | Effect | p-value |
| Sult2a2 | 11.7 | 1 | 0.8 | 0.008 | 1.4 | 0.019 | -12.4 | 0.000 | 11.6 | 0.000 |
| Sult3a1 | 11.7 | 2 | -1.8 | 0.174 | 4.5 | 0.000 | 3.2 | 0.000 | -0.4 | 0.595 |
| Cyp2b9 | 9.0 | 3 | 1.8 | 0.000 | 9.4 | 0.000 | -8.9 | 0.000 | 11.3 | 0.000 |
| Cyp3a41 | 4.1 | 4 | 1.5 | 0.006 | 2.2 | 0.000 | 1.9 | 0.001 | 1.9 | 0.001 |
| Cyp4a14 | 3.9 | 5 | 3.2 | 0.000 | 7.6 | 0.000 | -0.6 | 0.065 | -1.4 | 0.016 |
| Cyp2b13 | 3.5 | 6 | 3.1 | 0.000 | 5.0 | 0.000 | -0.7 | 0.216 | 9.8 | 0.000 |
| Cyp2b10 | 3.4 | 7 | 1.1 | 0.035 | 3.4 | 0.000 | 3.5 | 0.000 | 1.0 | 0.143 |
| Cyp4a10 | 3.3 | 8 | 4.1 | 0.000 | 7.9 | 0.000 | -3.7 | 0.000 | 4.9 | 0.000 |
| Fmo3 | 3.2 | 9 | 3.1 | 0.000 | 3.9 | 0.000 | 0.6 | 0.089 | -0.6 | 0.161 |
| Hao3 | 3.1 | 10 | 0.3 | 0.346 | 1.4 | 0.005 | -4.5 | 0.000 | 2.5 | 0.002 |
| Mt2 | 2.2 | 11 | 0.4 | 0.552 | 2.4 | 0.000 | -3.3 | 0.000 | 0.7 | 0.143 |
| Mt1 | 2.0 | 12 | 1.2 | 0.005 | 3.2 | 0.002 | -3.5 | 0.000 | -0.7 | 0.622 |
| Cyp2a4 | 1.5 | 13 | 0.7 | 0.109 | 1.8 | 0.000 | -0.7 | 0.098 | -1.4 | 0.020 |
| Ugt1a1 | 1.0 | 14 | -0.3 | 0.496 | 0.3 | 0.315 | -0.4 | 0.092 | -0.9 | 0.003 |
| Sult1e1 | 0.8 | 15 | 0.8 | 0.001 | 0.6 | 0.167 | -1.6 | 0.000 | 0.7 | 0.163 |
| Ugt1a5 | 0.7 | 16 | -0.7 | 0.090 | 0.1 | 0.807 | -0.5 | 0.177 | -1.1 | 0.002 |
| Cyp2c38 | 0.7 | 17 | 1.2 | 0.005 | 1.0 | 0.009 | 0.5 | 0.265 | 0.2 | 0.615 |
| NqoI | 0.7 | 18 | 0.4 | 0.071 | 1.1 | 0.016 | 0.0 | 0.905 | -1.1 | 0.009 |
| Mgst3 | 0.6 | 19 | -1.0 | 0.012 | -0.5 | 0.319 | -0.8 | 0.042 | -1.7 | 0.001 |
| Sult1d1 | 0.6 | 20 | 0.8 | 0.013 | 1.6 | 0.000 | 0.2 | 0.438 | -0.9 | 0.001 |
| Oatp1a4 | 0.4 | 21 | 6.1 | 0.000 | 6.0 | 0.000 | 1.5 | 0.005 | -0.2 | 0.542 |
| Gsta4 | 0.0 | 22 | 0.9 | 0.029 | 0.5 | 0.205 | -0.1 | 0.777 | 0.3 | 0.508 |
| Cyp1a1 | -0.1 | 23 | 0.5 | 0.211 | 0.5 | 0.134 | -0.3 | 0.632 | -1.2 | 0.006 |
| Sult1a1 | -0.1 | 24 | 1.0 | 0.055 | 0.5 | 0.433 | -0.4 | 0.215 | 0.2 | 0.544 |
| Cyp2a5 | -0.2 | 25 | 2.3 | 0.000 | 1.7 | 0.001 | -0.2 | 0.815 | -0.5 | 0.199 |
| Por | -0.3 | 26 | 2.7 | 0.001 | 3.5 | 0.000 | 0.1 | 0.787 | -0.5 | 0.340 |
| Aldh3a2 | -0.3 | 27 | 2.2 | 0.002 | 1.6 | 0.008 | 5.3 | 0.000 | 3.6 | 0.000 |
| Aldh4a1 | -0.4 | 28 | 1.9 | 0.000 | -0.3 | 0.483 | 1.5 | 0.001 | -0.6 | 0.165 |
| Gsta2 | -0.6 | 29 | 1.0 | 0.013 | 0.5 | 0.150 | 0.5 | 0.109 | -0.5 | 0.244 |
| Papss2 | -0.6 | 30 | 1.4 | 0.014 | 0.6 | 0.097 | 0.5 | 0.203 | -1.2 | 0.008 |
| Ugt2b34 | -0.8 | 31 | 2.0 | 0.000 | 0.8 | 0.006 | 0.8 | 0.058 | 0.6 | 0.020 |
| Fmo1 | -0.9 | 32 | 2.1 | 0.003 | 1.4 | 0.001 | 1.2 | 0.007 | 0.3 | 0.317 |
| Ugt1a6a | -1.0 | 33 | 2.0 | 0.001 | 0.9 | 0.043 | 1.4 | 0.009 | -0.2 | 0.693 |
| Gstt1 | -1.2 | 34 | 3.8 | 0.000 | 2.4 | 0.000 | 5.6 | 0.000 | 4.5 | 0.000 |
| Ugt2b5 | -1.7 | 35 | 1.1 | 0.149 | 0.4 | 0.402 | 1.6 | 0.000 | 0.1 | 0.930 |
| Fmo5 | -2.0 | 36 | 3.0 | 0.000 | 1.7 | 0.000 | 1.0 | 0.003 | -0.4 | 0.107 |
| Ugt2b35 | -2.0 | 37 | 0.1 | 0.856 | -1.5 | 0.015 | 1.5 | 0.003 | 0.3 | 0.225 |
| Ugt1a9 | -2.1 | 38 | 1.8 | 0.001 | -0.7 | 0.289 | 2.3 | 0.000 | -0.7 | 0.193 |
| Aldh6a1 | -2.2 | 39 | 2.3 | 0.000 | 0.6 | 0.126 | 2.6 | 0.000 | -0.8 | 0.182 |
| Oatp1a1 | -2.5 | 40 | 1.8 | 0.010 | 0.0 | 0.983 | 1.2 | 0.023 | -0.4 | 0.302 |
| Sult5a1 | -3.0 | 41 | -2.0 | 0.004 | -2.4 | 0.025 | 5.4 | 0.000 | 3.3 | 0.000 |
| Adh4 | -3.3 | 42 | 0.6 | 0.252 | -2.8 | 0.000 | 1.4 | 0.002 | -1.5 | 0.006 |
| Ces2c | -3.3 | 43 | 1.3 | 0.008 | -2.8 | 0.000 | 6.1 | 0.000 | 5.5 | 0.000 |
| Comt | -3.3 | 44 | 2.7 | 0.000 | -0.5 | 0.309 | 2.2 | 0.000 | 0.2 | 0.673 |
| Ugt2b1 | -3.4 | 45 | 1.7 | 0.005 | -1.0 | 0.097 | 1.7 | 0.004 | 0.1 | 0.830 |
| Cyp7b1 | -3.6 | 46 | 0.1 | 0.768 | -0.7 | 0.289 | 3.5 | 0.000 | -4.0 | 0.000 |
| Bcrp | -3.7 | 47 | 2.2 | 0.000 | -0.6 | 0.067 | 3.1 | 0.000 | 1.1 | 0.002 |
| Ces3a | -3.9 | 48 | 1.8 | 0.000 | -0.5 | 0.300 | 2.2 | 0.000 | 0.0 | 0.876 |
| Gstp2 | -4.3 | 49 | 2.0 | 0.001 | -0.6 | 0.130 | 1.1 | 0.007 | 0.7 | 0.078 |
| Gstp1 | -4.4 | 50 | 1.6 | 0.001 | -1.1 | 0.064 | 1.5 | 0.001 | 0.3 | 0.652 |
| Cyp2d9 | -5.5 | 51 | 1.5 | 0.014 | 0.0 | 0.946 | 7.0 | 0.000 | -3.2 | 0.000 |
| Cyp4a12 | -10.8 | 52 | 0.3 | 0.473 | 0.8 | 0.317 | 15.6 | 0.000 | -8.0 | 0.000 |

Legend to table:

Sex ratio is the log2 of the ratio of expression in female controls divided by male controls, using young adult UM-HET3 mice. Values above zero indicate higher level expression in female controls; values below zero are genes expressed more by male control mice.

Gene # indicates the order of the genes as shown in Figure 4.

Effect is the log2 of the ratio of treated to control mice of the indicated sex. Values above 0 indicate genes whose expression is increased by DR or rapamycin, and these are highlighted in red (if p < 0.05) or pink; values below zero indicate genes whose expression is diminished by the intervention, and these are highlighted in blue (if p < 0.05) or light blue.

p-values reflect t-test statistics calculated on raw Ct values from N = 6 treated and 6 control mice of each sex. The p-values are "nominal" in that they have not been adjusted for multiple comparisons.

**Supplemental Table S4: Characteristics of the assays used for the work shown in Figure 3.**

| **Analyte** | **Nominal sensitivity** | **Nominal CV** | **Our CV (median)** |
| --- | --- | --- | --- |
| FGF-21 | 10 pg/ml | 5.1% | 5.6% |
| Leptin | 0.2 ng/ml | 10% | 3% |
| Insulin | 0.12 ng/ml | 6.2% | 8.4% |
| IGF-1 | 0.02 ng/ml | 3.3% | 10.1% |
| T4 | 0.76 microgram/dl | 5.4% | 8.2% |

The table shows characteristics of the assay kits used for the hormone assays shown in Figure 3. Nominal sensitivity is as specified by the manufacturer of the kit. Nominal CV (coefficient of variation) is for intra-assay comparisons. Our CV is given as the median CV (intra-assay, for duplicates) of the set of samples evaluated.
